# A Diagnostically Challenging Case of Typical Hemolytic Uremic Syndrome Successfully Treated With Eculizumab Therapy

**DOI:** 10.7759/cureus.84362

**Published:** 2025-05-18

**Authors:** Adarsh Jha, Georgette Nader, Sumugdha Rayamajhi, Muhammad Hamdan

**Affiliations:** 1 Internal Medicine, Michigan State University College of Human Medicine, East Lansing, USA; 2 Hematology and Oncology, Sparrow Hospital, Lansing , USA

**Keywords:** bleeding per rectum, hemolysis, hemolytic uremic syndrome, microangiopathy, schistocytes

## Abstract

Hemolytic uremic syndrome (HUS) is a thrombotic microangiopathy that induces microvesicular injury and occlusion and often results in acute renal failure. Atypical HUS is life-threatening and often progresses to end-stage renal disease (ESRD). However, distinguishing between typical and atypical HUS can be challenging due to comorbid conditions and/or laboratory delays. We present a severe case of typical HUS involving a series of complications requiring multidisciplinary care, successfully treated with eculizumab therapy.

## Introduction

Hemolytic uremic syndrome (HUS) is a thrombotic microangiopathy that results in injury and occlusion of microvessels. Organ damage through vessel occlusion and thrombocytopenia predominantly affects the kidney, leading to acute renal failure [1]. This triad of microangiopathic hemolytic anemia, thrombocytopenia, and acute kidney injury uniquely characterizes HUS [2]. HUS can be further distinguished into typical and atypical forms.

Patients with typical HUS (tHUS) and atypical HUS (aHUS) exhibit the same classic triad of microangiopathic hemolytic anemia, thrombocytopenia, and acute kidney injury. However, the acquisition, pathophysiology, and treatment vary. tHUS occurs more commonly and predominantly affects children more than six months of age. tHUS results from an infection with a Shiga toxin or Shiga-like toxin bacteria, such as *E. coli* or invasive pneumococci [1,3]. Transmission is generally foodborne, with patients often presenting following one week of bloody diarrhea. Treatment is primarily supportive [3,4]. Whereas, aHUS is a rare, life-threatening form of HUS that occurs in 0.23 to 1.9 per million population for all age groups [5]. aHUS has a predilection towards children (2.2 to 9.4 per million population affected) and adult females [5,6]. Around 70% of cases are associated with an underlying genetic or acquired defect in the complement; however, a genetic mutation is not required for diagnosis [7,8]. Unlike tHUS, aHUS is not associated with a Shiga toxin-producing *E. coli* (STEC) infection. Common triggers include pregnancy, malignancy, medications, autoimmune conditions, organ transplant, infection, surgery, etc. [8]. Once diagnosed, however, prompt management is crucial, as prognosis is poor. aHUS has a 10 to 15% mortality rate during the acute phase, and within one year of diagnosis, 50% of patients will require dialysis and/or progress to end-stage renal disease (ESRD) [9,10]. The case described illustrates the diagnostic and therapeutic challenges of managing HUS.

## Case presentation

**Figure 1:**
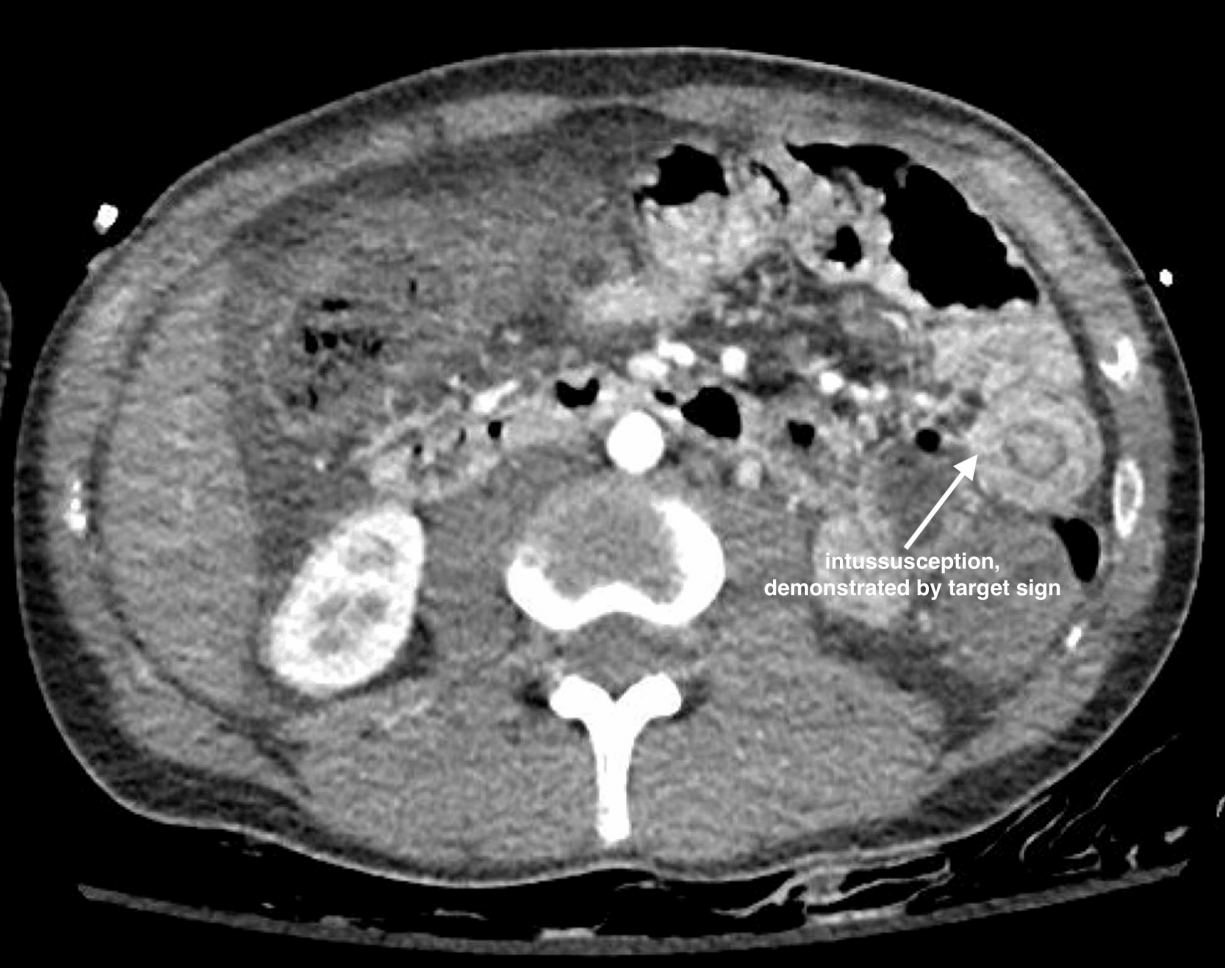
Axial CT angiogram showing intussusception in the left upper quadrant

**Figure 2:**
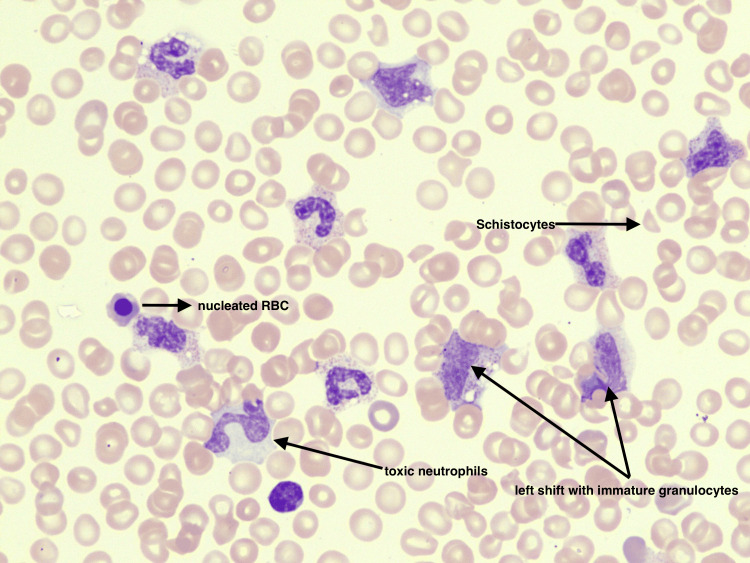
Schistocytes, nucleated red blood cells, left shift with immature granulocytes, and toxic neutrophils, findings consistent with a leukoerythroblastic reaction and microangiopathic hemolytic anemia (MAHA)

A 26-year-old male with a past medical history of bipolar disorder, autism, attention deficit hyperactivity disorder, oppositional defiant disorder, and pervasive developmental disorder presented to the hospital with a chief complaint of diarrhea associated with rectal bleeding due to perianal itching. Upon presentation, caregivers reported the patient to be lethargic, which was considered to be altered from baseline. On presentation, he was vitally stable. Table 1 depicts labs upon presentation and throughout the hospital course. The CT brain was negative for acute pathology. CT angiogram of the abdomen was positive for diffuse pancolonic wall thickening with wall enhancement suggestive of infectious or inflammatory colitis, moderate volume abdominal and pelvic ascites, and small bowel intussusception seen in the left upper quadrant (Figure 1). The physical exam was positive for abdominal distension with mild tenderness in the left lower quadrant. General surgery and gastroenterology were consulted, and recommended medical management. The patient was empirically treated with intravenous (IV) ceftriaxone 1g daily and IV metronidazole 500mg three times daily. Blood cultures resulted as positive for methicillin-sensitive *Staphylococcus aureus* (MSSA) bacteremia, for which the patient was treated with IV cefazolin 1 g daily. As the hospitalization progressed, the patient became more lethargic and self-abusive and required physical restraints due to resistance to care. Thus, although the stool cultures were ordered upon admission, samples were difficult to obtain until later in the hospitalization. Peripheral blood smear (PBS) on day three of admission was negative for schistocytes; however, PBS on day four showed significant leukoerythroblastosis, a significant left shift of the white cell with toxic granulation, and significant vacuolation. The red cells were positive for a few fragmented red blood cells/schistocytes, nucleated red blood cells, and polychromasia (Figure 2). These findings were consistent with reactive bone marrow, a leukoerythroblastic picture, and microangiopathic hemolytic anemia. Despite supportive therapy, hemoglobin and platelet levels continued to drop, requiring three units (U) of packed red blood cells (PRBC), two U of platelets, and four U of plasma. Due to high suspicion for aHUS, hemophagocytic lymphohistiocytosis (HLH), or thrombotic thrombocytopenic purpura (TTP), additional workup was done (Table 2). HIV, hepatitis C, and hepatitis B were negative. Shiga toxin later came back as positive, and antibiotics were discontinued to reduce toxin release. On day six of admission, the patient was transferred to the intensive care unit (ICU) for hemodialysis requirements. After multidisciplinary discussions, we opted to initiate eculizumab. On day six, the patient was given HiB, meningococcus, and pneumococcus vaccines and started on eculizumab 900 milligrams, with two weekly maintenance doses. The ICU course was complicated by agitation requiring sedation, acute respiratory distress syndrome (ARDS) requiring mechanical ventilation, and hemodialysis. While under sedation, the patient underwent a bone marrow biopsy, which was not suggestive of myeloid or lymphoid neoplasm. Following the initiation of eculizumab, the patient received one additional dose of 900 milligrams a week later. With this management strategy, the patient’s physical and mental status returned to baseline, and he was discharged home in the absence of long-term dialysis requirements. 

**Table 1:** Hospital course

Laboratory Component	Normal Value	Hospital Day 1	Hospital Day 4	Hospital Day 6	Hospital Day 8	Hospital Day 10	Hospital Day 14	Hospital Day 28
Creatinine (Cr)	0.6-1.40 mg/dL (Patient's baseline: 1.2-1.3)	1.21	1.81	5.3	4.01	3.9	3.66	1.71
Glomerular Filtration Rate (GFR)	>60 mL/min	>60	52	14	20.0	21.0	22.0	56.0
Lactate	0.2-1.8 mmol/L	5.2	1.5	2.1		1.5		
White Blood Cells (WBC)	4-12 x10^3^/uL	21.9	44.7	29.6	28.5	26.8	11.9	6.6
Hemoglobin (Hgb)	12.6-16.5 g/dL	15.5	7.6	6.3	8.1	7.2	8.5	7.8
Haptoglobin	36-195 mg/dl		<6				52.0	
Absolute Reticulocyte Count	0.03-0.08		0.044				0.272	
Platelet Count	150-400 x10^3^/uL	97	23	43	86.0	188.0	279.0	398.0
Ferritin	14-224 ng/mL		2305					
Lactate Dehydrogenase (LDH)	100-225 U/L			2248	669.0	558.0	479.0	190.0
D-dimer	0-0.5 mg		22.9	6.32	3.82	3.11		
Fibrinogen	150-459 mg/dL		381	241	364.0	396.0		
Activated Partial Thromboplastin Time (aPTT)	21-31 s		39.8	31.6	32.5	33.3		
Prothrombin Time	9.5-12.1 s		18.4	14.9	10.9	11.6		

**Table 2:** Protein levels

Protein	Patient's Value	Normal Values
Complement Protein C3	34	79-152 mg/dL
Complement Protein C4	<4	16-38 mg/dL
Total Complement/Ch50	<17	>41
Plasma Protein S Activity	15%	63-140%
Functional Protein S/ C1Q	23	34-63 U/mL
Functional Protein C	30%	73-180%
C5 Complement Antigen	17.7	10.6-26.3
ADAMTS13	51	66.8%

## Discussion

In the acute setting, clinical differentiation between tHUS and aHUS poses a unique challenge to clinicians. As both patients can present with elevated creatinine progressing to renal failure, cardiovascular, central nervous system, and/or gastrointestinal symptoms. Furthermore, prompt differentiation between critical illnesses of HUS, TTP, disseminated intravascular coagulation (DIC), and sepsis is crucial to ensuring appropriate management. Our patient posed a particularly unique challenge as he presented with three out of four common symptoms: mentation change, renal failure, intussusception, and diarrhea that persisted despite supportive therapy. Initial workup was suggestive of intravascular and microangiopathic hemolysis (elevated lactate dehydrogenase (LDH), reticulocyte count, low haptoglobin, schistocytes present on PBS), renal failure (elevated Cr), and hypercoagulability (low protein C and S). However, distinguishing TTP, aHUS, and tHUS was difficult due to overlapping pathways between the complement and hemostatic factors. With a moderately reduced ADAMTS13 of greater than 10%, it reduced the likelihood of hereditary or acquired TTP; however, it could not definitively exclude TTP. Other differentials of consideration were DIC and HLH, which have an ICU mortality rate of 57% [11]. Fibrinogen levels were above normal and less indicative of DIC, and bone marrow biopsy was negative for HLH or malignancy. Distinguishing between aHUS and tHUS thus relied on additional laboratory studies, which were delayed in processing. While awaiting additional laboratory studies, our patient failed to improve with supportive therapy and experienced a notable decline in mentation and respiratory status. Based on his worsening clinical presentation and the above pending laboratory results, treatment with hemodialysis and eculizumab was initiated simultaneously.

Eculizumab is a recombinant, humanized, monoclonal immunoglobulin G antibody that blocks the cleavage of C5 to C5b, thus preventing the formation of the proinflammatory peptide C5b and the cytotoxic membrane attack complex C5b-9 (MAC) [2]. Eculizumab is approved for use in aHUS and paroxysmal nocturnal hemoglobinuria (PMH). However, little data is available for its use in tHUS. A few case reports have reported significant neurological improvement with the use of eculizumab in HUS [12,13,14]. A retrospective analysis by Pape et al. showed a temporal relationship of eculizumab use in patients with tHUS. Such that earlier treatment initiation resulted in rapid neurological improvement; meanwhile, late initiation resulted in progression of multiorgan involvement [12]. Our case lends support to the use of eculizumab in the treatment of severe tHUS. Simultaneous use of eculizumab with hemodialysis was chosen due to the minimal amount of anticipated loss of eculizumab based on its large molecular weight. Treatment was initiated on hospital days six and 14. Within 24-48 hours, the patient had a rapid improvement in neurological standing. He was communicative, answering questions appropriately, calm, and noncombative. Additionally, laboratory and renal function dramatically improved over the hospitalization, as illustrated in Table 1.

## Conclusions

In conclusion, this case highlights a fulminant presentation of tHUS in a young adult male, which was initially difficult to distinguish from aHUS and TTP due to multi-organ dysfunction and a declining mental status. The patient’s condition did not improve with conventional supportive therapy alone, but he showed a rapid and remarkable recovery after the initiation of the complement inhibitor eculizumab in conjunction with hemodialysis. This outcome supports the consideration of eculizumab in select, severe cases of tHUS where clinical deterioration is rapid and the diagnosis is delayed or uncertain. More broadly, our experience suggests that early use of complement blockade may benefit patients with fulminant HUS presentations even when the final diagnosis is typical (Shiga toxin-mediated) HUS by controlling the thrombotic microangiopathy and preventing irreversible organ damage. This case highlights the importance of maintaining a high index of suspicion and being willing to employ targeted complement-inhibiting therapy in life-threatening HUS cases to improve patient outcomes in clinical practice.
